# Experimental Validation of Motor Primitive-Based Control for Leg Exoskeletons during Continuous Multi-Locomotion Tasks

**DOI:** 10.3389/fnbot.2017.00015

**Published:** 2017-03-17

**Authors:** Virginia Ruiz Garate, Andrea Parri, Tingfang Yan, Marko Munih, Raffaele Molino Lova, Nicola Vitiello, Renaud Ronsse

**Affiliations:** ^1^Center for Research in Mechatronics, Institute of Mechanics, Materials, and Civil Engineering, Université catholique de Louvain, Louvain-la-Neuve, Belgium; ^2^Institute of Neuroscience, Université catholique de Louvain, Brussels, Belgium; ^3^“Louvain Bionics”, Université catholique de Louvain, Louvain-la-Neuve, Belgium; ^4^The BioRobotics Institute, Scuola Superiore Sant’Anna, Pontedera, Pisa, Italy; ^5^Laboratory of Robotics at the Faculty of Electrical Engineering, University of Ljubljana, Ljubljana, Slovenia; ^6^Don Carlo Gnocchi Foundation, Florence, Italy

**Keywords:** bio-inspired, primitives, control, exoskeleton, orthosis

## Abstract

An emerging approach to design locomotion assistive devices deals with reproducing desirable biological principles of human locomotion. In this paper, we present a bio-inspired controller for locomotion assistive devices based on the concept of motor primitives. The weighted combination of artificial primitives results in a set of virtual muscle stimulations. These stimulations then activate a virtual musculoskeletal model producing reference assistive torque profiles for different locomotion tasks (i.e., walking, ascending stairs, and descending stairs). The paper reports the validation of the controller through a set of experiments conducted with healthy participants. The proposed controller was tested for the first time with a unilateral leg exoskeleton assisting hip, knee, and ankle joints by delivering a fraction of the computed reference torques. Importantly, subjects performed a track involving ground-level walking, ascending stairs, and descending stairs and several transitions between these tasks. These experiments highlighted the capability of the controller to provide relevant assistive torques and to effectively handle transitions between the tasks. Subjects displayed a natural interaction with the device. Moreover, they significantly decreased the time needed to complete the track when the assistance was provided, as compared to wearing the device with no assistance.

## Introduction

Assistive locomotion devices have attracted a growing focus of attention in the recent years. This increasing interest emerged mainly from three incentives. First, most developed countries have a graying population. Demographic evolution forecasts that, in 40 years from now, about 21% of the population in Europe will be older than 60 years (UN Department of Economic and Social Affairs, Population Division, 2013). This population sector not only displays a natural decline of locomotor skills but also shows a higher incidence of diseases or accidents causing permanent gait disorders. For example, cardiovascular diseases increase the risk of lower limb amputation, while stroke is often associated with hemiparesis (Macciocch et al., 1998; Verghese et al., 2006). Therefore, technologies supporting a sustainable aging population are urgently requested to improve the autonomy of these patients and keep them active in society. Second, assistive devices are of relevant interest for younger patients with lifelong disabilities who wish to enhance their locomotion skills. For instance, recent progresses allowed spinal cord injured patients to “be walked” within an exoskeleton so that they (re)experience bipedal standing and walking (Strausser and Kazerooni, 2011; Esquenazi et al., 2012). Third, exoskeletons are also being currently developed as power augmentation devices in industrial and military applications.

An extensive review of the main assistive locomotion devices and their control strategies is provided in Díaz et al. (2011), Chen et al. (2013), and Yan et al. (2015a) for these three families of applications. These reviews reveal that there is still a need to develop effective control strategies for these devices. The ideal controller should manage the human–robot interactions while evolving in complex environments (unstructured grounds, slopes, stairs, etc.) with minimum cognitive effort for the user. This is especially critical for the elderly, who are more prone to rapidly refusing a cognitively demanding robot.

One emerging control approach deals with exploring bio-inspired assistive models (Ijspeert, 2014). These controllers aim to adopt some locomotion principles and mechanisms identified in humans and transpose them to artificial devices. Bio-inspiration targets both to reproduce desirable characteristics of human locomotion, e.g., robustness and adaptability, and to generate an intuitive interaction between the user and the artificial leg.

For example, a well-developed strategy for exoskeleton control deals with decoding electromyographic signals (EMGs) and producing torques being proportional to them (He and Kiguchi, 2007; Hassani et al., 2013), i.e., like a biological muscle would do. However, this approach suffers from issues due to the placement complexity of the sensory apparatus and to the required computationally greedy signal processing. Moreover, EMG recordings being intrinsically noisy and difficult to scale, they require thorough calibration and processing for each user and even each session (Türker, 1993).

Another bio-inspired approach relies on the so-called central pattern generators (CPGs). CPGs are neural networks located in the spinal cord of mammals that are able to produce rhythmic outputs without receiving cyclic input signals (Ijspeert, 2008; McCrea and Rybak, 2008). Said differently, they are considered to be the biological clock for rhythmic movements. Several controllers were developed using adaptive oscillators (AOs) as mathematical tools to emulate the role of CPGs by extracting the periodic parameters of locomotion. Given a periodic input signal, an AO is capable of tracking its phase and frequency by capturing these features in state variables. When the periodic signal is related to a gait pattern, the estimated phase and frequency are the ones associated with the performed locomotion task. These parameters could be mapped to desired assistive torques obtained from literature data or previously recorded sessions (Lenzi et al., 2013). This requires however to add more profiles for each locomotion task that the device should assist. Other contributions exploited the potential of AOs to learn and synchronize to a particular pattern. Using virtual impedance fields, the user can be “attracted” to the predicted future position of his/her own joints, thus providing assistance. While this approach has been widely investigated for assisting ground-level walking (Ronsse et al., 2011; Giovacchini et al., 2015), it has never been validated for assisting different locomotion-related activities. Moreover, it is prone to face difficulties during transitions from one locomotion task to the next, i.e., when there are significant changes in the gait pattern.

Handling transitions is a compelling topic, and most existing controllers for wearable exoskeletons mainly focus on the walking task. Other maneuvers such as stair climbing and descending are far less explored. Moreover, the few studies exploring ascending and descending stairs assistance usually disregard the management of transitions between the locomotion tasks (Mori et al., 2005; He and Kiguchi, 2007; Yeh et al., 2010; Ekelem et al., 2015). Some research projects developed exoskeletons allowing different tasks to be performed one after the other. However, they often required the subject to stop before starting a new locomotion task. This means that, even if the controllers can cope with different locomotion tasks by using the user’s intention as input, most of them disregard the possibility to generate continuous and smooth transitions between these tasks. For example, ReWalk embeds a wrist-pad interface to select the desired locomotion task (Esquenazi et al., 2012; Murtagh, 2015). One exception is the knee exoskeleton TUPLEE (Fleischer and Hommel, 2008), which uses a controller acting as a torque amplifier based on EMG. It is thus able to deal with smooth transitions between tasks (sit to stand, walk to stairs, etc.). Another example is the power enhancement RoboKnee (Pratt et al., 2004), a knee exoskeleton providing an assistive torque computed from the ground reaction forces. However, to the best of our knowledge, no report about the device behavior during the transition phases is available in the literature yet.

In this contribution, we develop an assistive controller for several locomotion tasks—and transitions between them—combining AOs with motor primitives. Biologically speaking, motor primitives lie on a network of spinal neurons that activates a determined set of muscles (Degallier and Ijspeert, 2010). Consequently, a low-dimensional set of basic signals (the so-called primitives) can provide a high-dimensional set of muscle stimulations, pending a proper recombination through the spinal weights. Primitives offer the key advantage of reducing the control dimensionality: a small set of basic signals can produce a much higher dimensional set of stimulations (Degallier and Ijspeert, 2010; Gonzalez-Vargas et al., 2015). Moreover, primitives support inter-limb coordination, since many muscles are stimulated by the same primitives through simultaneous activation. Finally, the use of the exact same set of primitives for different locomotion tasks and a low-dimensional control to activate them reduces the computational load. Soft transitions between different tasks can further be obtained by smoothly switching the corresponding primitive weights. In a recent study, we explored the concept of primitive-based assistance with level ground walking experiments using an assistive pelvis exoskeleton (Ruiz Garate et al., 2016a). In the present paper, we extend the validation to a wider motor repertoire including stairs climbing and descending, and also assistance to the distal joints (knee and ankle) of one leg. Moreover, we explore the performance of the assistive strategy during continuous transitions between the different locomotion tasks. This work thus provides the full validation of the controller capabilities. All considered tasks and the transitions between them were tested with healthy participants. Simultaneously, subjects received multi-joint assistance to the three leg joints through a unilateral leg exoskeleton. Importantly, the controller was validated in out-of-lab environment, thus with more challenging settings than the typical setup of experiments investigating locomotion assistance, i.e., a speed-controlled treadmill.

In the following sections, first, the motor primitive-based controller is introduced. Then, its validation through a series of experiments is presented. Next, results from these experiments are displayed and discussed. Finally, the paper ends with a conclusion. A preliminary version of the present paper has already been published in a conference proceeding (Ruiz Garate et al., 2016b). The present version reports more methodological details and more results to validate the suggested approach.

## Materials and Methods

In this section, the control system based on human-like motor primitives is described (see Section “Motor Primitive-Based Controller”). Then, the experimental protocol to validate this proposed primitive-based controller is outlined (see Section “Experimental Validation”).

### Motor Primitive-Based Controller

Motor primitives form a low-dimensional set of signals that can generate a higher dimensional set of stimulations, both for many muscles and for different locomotion tasks. In humans, these stimulations activate a set of muscle–tendon units (MTUs) providing the corresponding joint torques. These torques eventually produce movements such as ground-level walking, ascending stairs, or descending stairs.

Our controller builds upon the emulation of this process by generating sagittal reference joint torques for locomotion assistance. An assistive device can then be used to transfer these torques—scaled as a function of the required level of assistance—to a human user.

**Figure 1:**
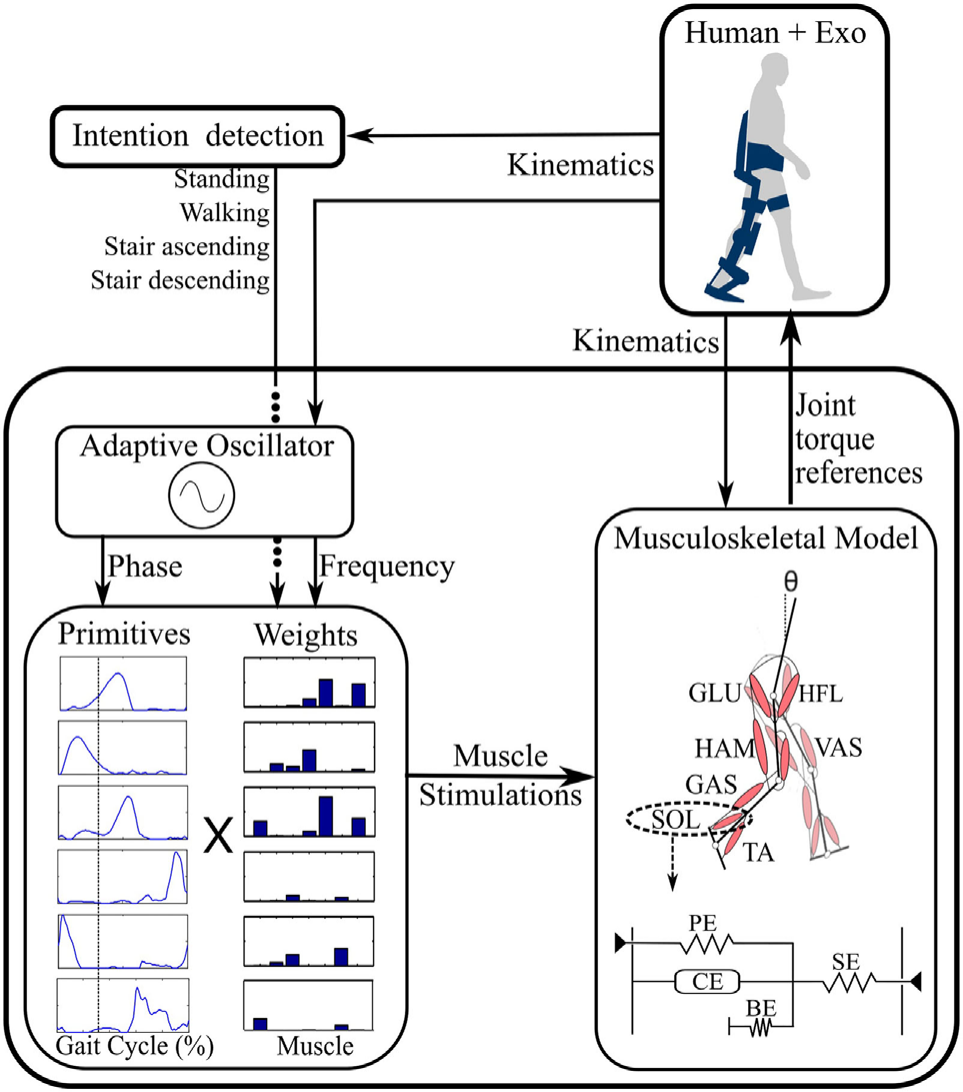
**General control diagram**. From the detected locomotion task and kinematics, primitives are combined through the corresponding weights, generating muscle stimulations. These stimulations activate a seven muscle–tendon unit (MTU) model further generating the reference joint torques. A typical Hill-type MTU is outlined below the musculoskeletal model [adapted from Geyer and Herr (2010)]. See the text for the definition of the different acronyms.

A general diagram of the developed control strategy is depicted in Figure 1. The intention detection block uses the measured kinematics to decode the locomotion task being performed. Moreover, an AO is used to extract the gait frequency and phase. Based on these detected task and gait features, primitives are then combined using appropriate weights. This combination generates a set of artificial muscle stimulations that activate a musculoskeletal model (which, in our case, is virtual, i.e., simulated) further generating assistive joint torques. These different components of the controller are described hereafter.

#### Musculoskeletal Model

The musculoskeletal model is implemented based on the one developed in Geyer and Herr (2010), comprising seven virtual MTUs per leg. Each unit captures the following leg muscle groups: hip flexor muscles (HFL), gluteus (GLU), hamstring (HAM), vastus (VAS), gastrocnemius (GAS), tibialis anterior (TA), and soleus (SOL) (Figure 1).

These MTUs are modeled as Hill-type muscles (Figure 1 bottom right). The force ***F***_**m**_ being generated by a muscle results from the interaction between a series elastic element, a parallel elastic element, a buffer elasticity preventing the muscle from collapsing, and an active contractile element (CE) (Geyer and Herr, 2010). The generated muscle forces are then converted into muscle torques through geometrical relationships: **τ**_**m**_ = ***r***_**m**_***F***_**m**_, where ***r***_**m**_ corresponds to the lever arm of the muscle attachment point (Geyer and Herr, 2010).

Finally, the musculoskeletal model computes the torques to be delivered at the leg sagittal joints (hip, knee, and ankle) as a function of the torque provided by each muscle:

$$\tau_{\mathrm{HIP}}=\tau_{\mathrm{HAM}}+\tau_{\mathrm{GLU}}-\tau_{\mathrm{HFL}}+\tau_{\mathrm{lH}}$$

$$\tau_{\mathrm{KNEE}}=\tau_{\mathrm{VAS}}-\tau_{\mathrm{GAS}}-\tau_{\mathrm{HAM}}+\tau_{\mathrm{lK}}$$

$$\tau_{\mathrm{ANKLE}}=\tau_{\mathrm{SOL}}+\tau_{\mathrm{GAS}}-\tau_{\mathrm{TA}}+\tau_{\mathrm{lA}}$$

where **τ**_**lH**_, **τ**_**lK**_, and **τ**_**lA**_ are torques preventing the hip, knee, and ankle from reaching their physical limits (Geyer and Herr, 2010). Note that these relationships clearly capture the bi-articular nature of GAS and HAM, since they produce torques across two joints.

Numerical parameters defining MTU and skeleton geometry were taken from the ones in Geyer and Herr (2010), pending some adaptations to the subject’s anthropometry (see Section “Motor Primitives”).

#### Motor Primitives

The MTU active component is the above mentioned CE, which produces a force being proportional to the muscle stimulation. From a biological viewpoint, these muscle stimulations would correspond to the EMG signals activating the corresponding muscles.

In the proposed framework, these virtual stimulations are generated by a low-dimensional set of primitives. This section reports how these primitives were constructed from literature data providing reference kinematics and dynamics (Table 1). For this purpose, an inverse model of the musculoskeletal model outlined in Section “Musculoskeletal Model” was created and scaled as a function of the anthropometry reported for each data set. Originally, numerical parameters of the MTUs and of the skeleton geometry were taken from the ones in Geyer and Herr (2010). The adaptations involved that the optimal and slack lengths of the muscles and the muscle attachment lever were scaled proportionally to the height of the participants of the exploited data (Table 1), whereas the maximal isometric force was scaled proportionally to the square of their height. While rather simplistic, this approach provided a handy normalization across the different dataset as a function of the corresponding subjects’ anthropometry, following also some biological observations (Samaras, 2007).

**Table 1:** **Reference data for the primitives generation**.

Reference	Task	Cadence (cycles/s)	Number of subjects
Winter (1991)	Walking	0.72	19
		0.88	19
		1.03	17
Wang et al. (2012)	Walking	0.82	5
		0.91	5
		0.98	5
		1.04	5
Koopman and van Dijk (2010)	Walking	0.6	4
		0.7	5
		0.8	8
		0.9	3
Bradford and Winter (1988)	Stairs ascend	X	3
	Stairs descend	X	3
Riener et al. (2002)	Stairs ascend	X	10
	Stairs descend	X	10

Moreover, as the model defined in Geyer and Herr (2010) focused on a particular walking task, further adaptations were necessary to produce muscle forces of larger magnitude for ascending and descending stairs. Concretely, the optimal length of several muscles was further scaled after the anthropometry adjustments: HFL, HAM, TA, and SOL lengths were increased 1.5 times, whereas the one of GAS was increased 2.5 times. These changes were found empirically in order to minimize the set of parameters to be changed. Once adjusted, these values were then kept invariant for all tasks.

In order to obtain the muscle stimulations from the kinematics and dynamics data, first, muscle forces were computed. However, this problem is redundant, since there are more muscles than joints for each leg. A unique solution was obtained by minimizing $\left\|\frac{f}{f_{\max}v_{\max}}\right\|$, while satisfying **τ** = *L* ⋅ *f* + **τ**_**l**_; ***f*** ≥ **0**; ***f*** ≤ ***f***_**max**_, where ***f*** is the vector of muscle forces, ***L*** is the matrix of lever arms (***r***_**m**_) projecting muscle forces into joint torque, and **τ**_**l**_ are torques preventing joints from reaching their physical limits. ***f***_**max**_ and ***v***_**max**_ capture the maximum isometric force and maximum contractile velocity of the muscles, respectively. Minimizing the normalized muscle force was performed in order to be biologically relevant with the many observations reporting that humans tend to do so (see, e.g., Todorov and Jordan, 2002 and Todorov, 2009). Moreover, this particular metric turned to provide the smallest residual stimulation errors with respect to known stimulation patterns, i.e., the ones in Geyer and Herr (2010). Afterward, an inverse model of the muscle dynamics presented in Geyer et al. (2003) and Geyer and Herr (2010) was applied to retrieve the stimulations from the forces.

The stimulations obtained from this inverse model were then normalized as a function of the duration of the gait cycle, so that the normalized duration ranged between 0 and 100% with 0 and 100% being coincident to two successive foot strikes^1^ of the corresponding leg. Moreover, in order to simplify the primitive extraction process, the stair stimulations obtained from Bradford and Winter (1988) and Riener et al. (2002) were averaged (normalized averaging, as a function of the number of subjects from both sources), and the walking ones (Winter, 1991; Koopman and van Dijk, 2010; Wang et al., 2012) were grouped and also averaged (normalized averaging, as a function of the number of subjects from the three sources) in five bins of walking cadences, i.e., [0.6–0.69], [0.7–0.79], [0.8–0.89], [0.9–0.99], and [1.0–1.09] cycles/s. In sum, from the data in Table 1, a high-dimensional set of seven muscle stimulations was obtained for seven different conditions: the five different walking cadences, stair ascending, and stair descending. These stimulations could be considered as the dimensionless neural input.

From these stimulations, a lower dimensional set of motor primitives was obtained by using non-negative matrix factorization (NNMF) (Tresch et al., 1999; Bizzi and Cheung, 2013). Because NNMF may converge to local minima, the process was repeated 100 times and the one with the lowest residual error was kept as solution.

**Figure 2:**
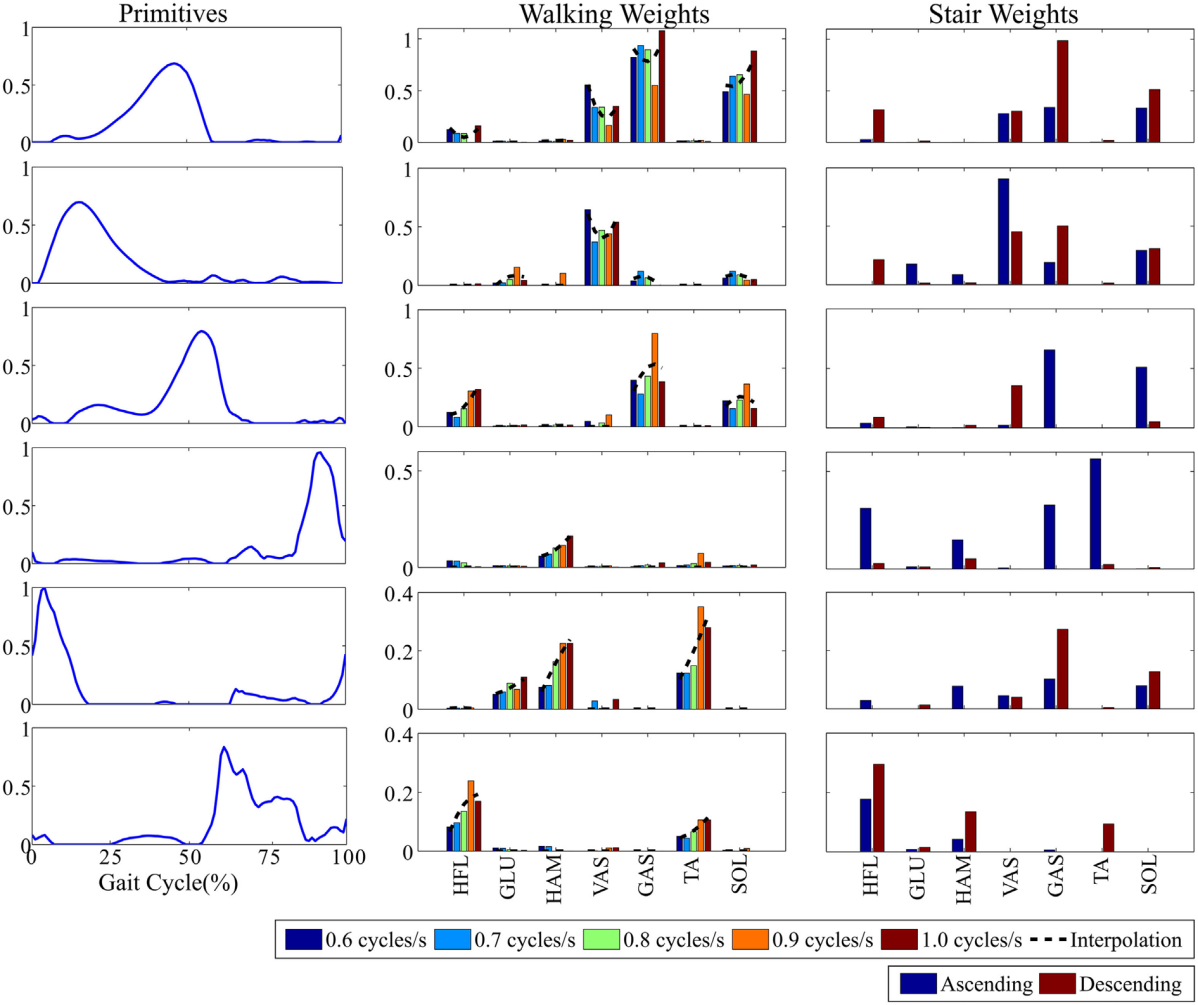
**The selected six primitives and their corresponding weights for the different walking cadences and for ascending/descending stairs**. Primitive weights across the different walking cadences were further interpolated by second-order polynomials, displayed in black dashed lines.

The NNMF process received as input the high-dimensional set of muscle stimulations and produced a set of primitives, accounting for a decreasing amount of the variance of the input signals. The first primitives were included in the low-dimensional set, up to reaching less than 4% of the normalized reconstruction error. This led to six primitives, accounting for more than 98% of the input variance (see Figure 2). This represents a much smaller amount than the original dimension of the input signals, i.e., 49. Figure 2 also provides the weights by which the primitives have to be multiplied to reconstruct the muscle stimulations. These weights are different for each task and each cadence (for walking only). In sum, six primitives could account for the seven muscle stimulations of three different tasks and several walking cadences.

In the case of the walking task, the five cadence-dependent weights obtained after the decomposition process were interpolated by second-order polynomials (see Figure 2). These polynomials were used to compute the weights for cadences ranging between 0.5 and 1.1 cycles/s, thus encompassing the learning range. Finally, when the learned cadence was below 0.3 cycles/s and above 1.3 cycles/s (i.e., clearly out of the learning range), the weights were forced to 0. A cadence out of these limits would thus produce no stimulation. In the intermediate ranges, i.e., [0.3–0.5] and [1.3–1.5] cycles/s, linear interpolations were used to guarantee the continuity of the weights evolution between both former cases.

#### Adaptive Oscillator

The combined primitives produce virtual muscle stimulations being modulated as a function of the gait phase and cadence. Therefore, it was necessary to estimate both the gait frequency (to properly modulate the primitive weights) and the instantaneous phase (to generate the primitives in a coordinated timing). Frequency and phase were estimated online by means of an AO (Righetti et al., 2006; Ronsse et al., 2013), using the right hip angle as input (see also Section “Experimental Setup”). The accuracy of the gait phase detection was further augmented by implementing a smooth phase reset mechanism at the instant of foot strike, according to the method developed in Yan et al. (2017). In the reported experiment, the phase reset parameters were tuned in order to achieve the correction of the detected phase error within one gait cycle. As a consequence, the estimated phase was the time-scaled reference of the gait cycle (increasing from 0 to 2π which corresponds to 0–100% of the total gait cycle duration), being 0% the landmark of foot strike.

#### Intention Detection

The intention detection block is in charge of detecting the onset of locomotion and of classifying the performed activity among walking, stair ascending, or stair descending tasks. This classification is necessary in order to select the appropriate weights for combining the primitives (see Figure 2). The intention detection employed in this controller was based on a real-time locomotion mode recognition algorithm presented in Yuan et al. (2015). This algorithm relies on a fuzzy-logic classifier operating on features extracted from hip joint angles and longitudinal position of the center of pressure at foot strike of the leading leg.

### Experimental Validation

**Figure 3:**
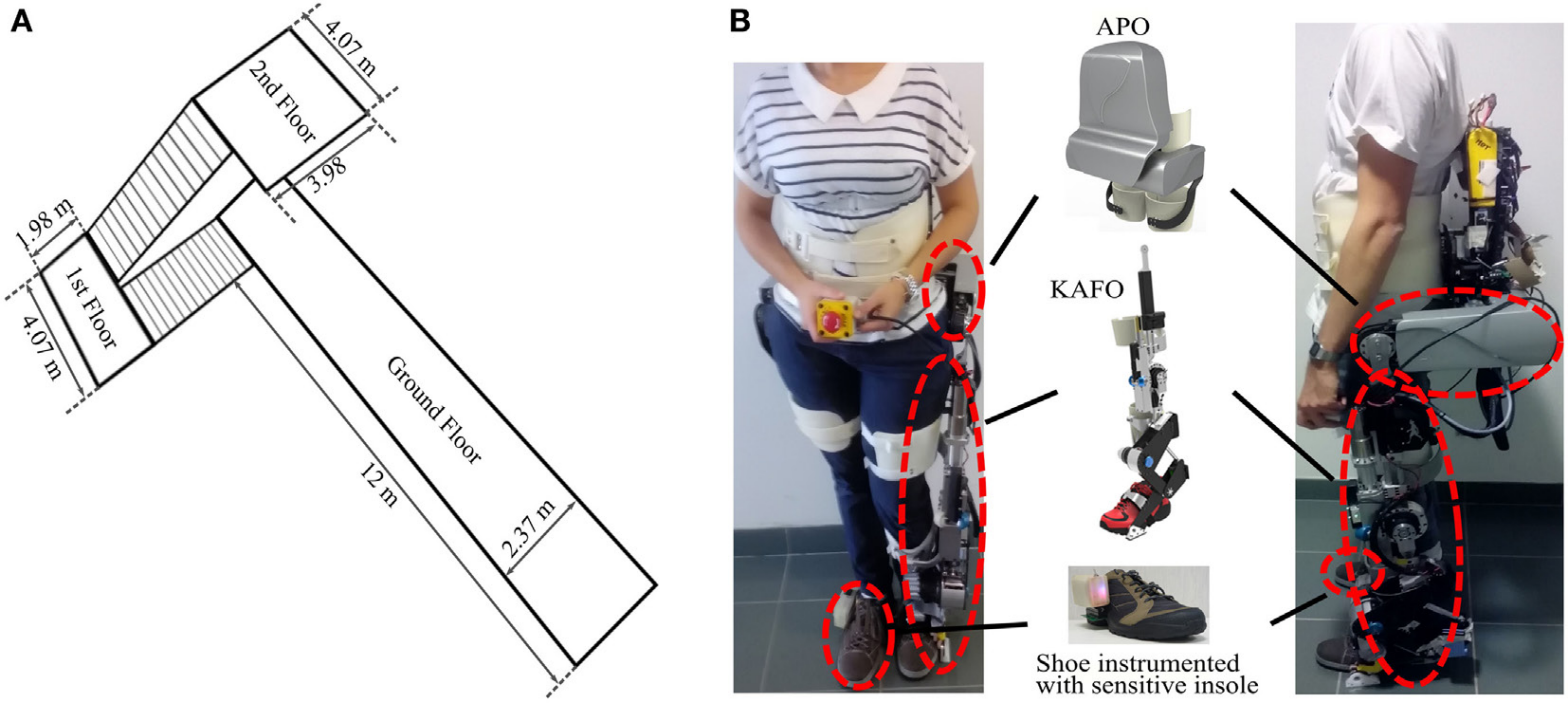
**(A)** Locomotion track followed by the subjects. The starting corridor had a length of 12 m and each set of stairs was composed of 14 steps. Steps were 31 cm wide and 17 cm high. **(B)** Two different subjects wearing the devices: left, S1—female, 66 kg, 1.65 m; right, S2—male, 65 kg, 1.84 m. The following components are visible: active pelvis orthosis (APO), knee-ankle-foot orthosis (KAFO), and the shoe instrumented with a custom pressure-sensitive insole.

This experiment aimed at proving the usability of the motor primitive-based controller to comply with three different locomotion tasks, i.e., walking, ascending stairs, and descending stairs, and to deliver appropriate assistive sagittal torques for the three leg joints of a powered hip–knee–ankle exoskeleton. Experimental activities were conducted at the premises of Don Carlo Gnocchi Foundation (Florence, Italy). A track was traced comprising ground-level walking, stair ascending, and stair descending, and covering all the possible transitions between these different locomotion tasks (Figure 3A).

This study was reviewed and approved by the Ethical Committee of the Fondazione Don Gnocchi (Florence, Italy), where the experiments were conducted. All subjects gave written informed consent in accordance with the Declaration of Helsinki.

#### Participants

Seven healthy participants—five males and two females, aged between 23 and 34—volunteered for the experimental sessions. The main characteristics of the participants are provided in Table 2. For these preliminary experiments, only healthy subjects were recruited because of the potential risk and difficulty of handling stair ascending/descending while being equipped with the devices described below.

**Table 2:** **Participants’ features and trials ordering**.

Subject	Gender	Age (years)	Weight (kg)	Height (m)	Trials sequence
*S1*	Female	34	66	1.65	AM—TM—NO
*S2*	Male	27	65	1.84	TM—AM—NO
*S3*	Female	31	73	1.75	AM—TM—NO
*S4*	Male	23	90	1.87	TM—AM—NO
*S5*	Male	25	60	1.72	AM—TM—NO
*S6*	Male	27	71	1.65	AM—TM—NO
*S7*	Male	26	60	1.72	TM—AM—NO

*NO, no orthosis; TM, transparent mode; AM, assistive mode*.

For each participant, the musculoskeletal model was again adapted as a function of their anthropometry. This adaptation followed the same principle as the one in Section “Motor Primitives.”

#### Experimental Setup

During the experiments, each participant was equipped with the following items: a bilateral active pelvis orthosis (APO), a knee-ankle-foot orthosis (KAFO) on the left leg, and a sensorized insole measuring the vertical reaction force under the right foot.

The APO is an advanced version of a previous laboratory prototype presented in Giovacchini et al. (2015). It consists of a bilateral hip orthosis for assisting hip movements in the sagittal plane during locomotion activities. The APO features a mechanical frame—adjustable to a wide range of anthropometries—endowed with two series elastic actuation (SEA) units designed to actively drive and comply with the hip flexion/extension movement (“Sistema di Attuazione per Ortesi di Anca”; Italian patent application no. FI/2015/A/000025). It is further equipped with absolute encoders providing the hip joint angles. These angles were used as input to the “Intention Detection” and “Adaptive Oscillator” blocks (see Figure 1). The system is wearable and tetherless, since the batteries and control electronics are integrated in a backpack. The control unit is a NI sbRIO 9632 (National Instruments, Austin, TX, USA) endowed with a real-time processor running at 100 Hz and a FPGA running at 1 kHz. The FPGA layer embeds the low-level closed-loop torque control for driving the active joints.

The KAFO (Yan et al., 2015b) was designed for providing assistance unilaterally to the left knee and ankle joints during locomotion. The flexion/extension of the knee is powered by a linear SEA, whereas the ankle is endowed with a variable stiffness actuator, the so-called MACCEPA (Van Ham et al., 2007; Jimenez-Fabian et al., 2015), characterized by a non-linear elasticity whose stiffness can be manually adjusted and with a continuous variable transmission which depends on the joint angular configuration. Though only the foot plantar/dorsiflexion is powered, a passive degree of freedom connected to a pair of dedicated shoes allows the user to freely perform inversion/eversion during gait. KAFO encoders further provide with joint angle measurements for the knee flexion–extension and ankle plantar/dorsiflexion. The KAFO and the APO can be handily connected to each other by means of a mechanical link in order to obtain a system capable of providing assistance to the hip, knee, and ankle joints in the sagittal plane. The KAFO is operated by the same control board as the APO, whereas its own batteries are integrated in the same backpack.

The insole measured the vertical component of the right foot reaction force and the load distribution (position of the center of pressure). This sensorized insole consists of a matrix of 64 optoelectronic transducers (Crea et al., 2014). This information was used to detect stance and swing phases of the right foot and reset the gait phase according to foot strike. Furthermore, the center of pressure was used as input to the “Intention Detection” block to classify between the different locomotion tasks.

Two participants with different anthropometries wearing the full setup are displayed in Figure 3B. During the experiments, the subjects were instructed to walk and take the stairs at their preferred speed.

During all trials, several safety rules were applied in order to guarantee a safe use of the device in an unstructured environment: (i) the assistance was only turned on after the device reached synchronization for the first time in the trial (phase estimation error at foot strike smaller than 0.63 rad, i.e., the 10% of the gait cycle); (ii) at the onset of delivering assistance, the controller gains smoothly increased until reaching their maximum value after 2 s; (iii) next, if the estimated phase error at foot strike happened to be larger than 10%, the assistance was decreased to 25% of its actual value; and (iv) if the detected maneuver corresponded to standing-still, all reference torques were set to 0 Nm, i.e., to be in the transparent mode (TM). Additionally, when assistance was applied, the reference torque was saturated to ±15, ±30, and ±25 Nm, for the hip, knee, and ankle, respectively.

During the experiments, the AO estimated the gait frequency and phase using the right hip angle as input. The rationale for selecting this signal was to prevent the dynamics of the assisted leg from influencing the synchronization process of the AO coupled to the right hip angle. If bilateral support is envisaged, future investigations will have to explore the impact of delivering assistance on the synchronization process (see Yan et al., 2017 as an example of the modifications in gait pattern induced by the interaction with an active exoskeleton). Consequently, we hypothesized the legs to follow a perfect anti-phase relationship across the different conditions, so that the left leg phase was obtained by adding a 50% shift to the one estimated from the right side.

In order to generate smooth and continuous transitions between the muscle stimulations (and thus the assistive torques), when switching from one locomotion task to another, a low pass filter was applied to the primitive weights with a time constant equal to 0.05 s.

#### Experimental Protocol

During the experimental session, the following trials were performed and analyzed:

- No orthosis mode (NO): the subject performed the track three times in a row without wearing the orthosis. This condition was used as a baseline to measure the time needed to complete the track without wearing the device.
- TM: the subject performed the track three times in a row while wearing the device. Assistive torques were not delivered, i.e., the powered orthosis was controlled to follow the intended movement of the person without exerting mechanical impedance on the user’s joints. Residual interaction torques were evaluated in previous experiments. In particular, the average root mean square of the residual torque under the TM condition within one walking cycle for a walking speed of 3.6 km/h was found to be equal to 0.51 ± 0.11, 1.28 ± 0.09, and 3.38 ± 0.19 Nm for hip, knee, and ankle joints, respectively. This condition aimed at measuring the impact of wearing the device on the subject’s kinematics, cadence, and time needed to complete the track.
- Assistive mode (AM) with assistance delivered with the motor primitives: the subject performed the track three times in a row while wearing the device and being actively assisted. Assistive torques were obtained by scaling the reference torque outputs from the controller to 15, 18, and 10% of their maximum for the hip, knee, and ankle, respectively. These levels were determined during pilot experiments in which participants performed a similar track (including all the tasks) several times. They were provided different assistive levels until reporting the ones being the most comfortable. The selected values for this experiment emerged as a consensus among the pilot participants. AM condition aimed at measuring the impact of the provided assistance on the subject’s kinematics, cadence, and time needed to complete the track. Since the subjects wore the KAFO only on the left leg, knee, and ankle joints were only assisted for this leg. At the level of the hip, only the left side was assisted as well, so that the right side was controlled to be transparent. Consequently, no joint on the right side received assistance.

TM and AM trials were randomized among subjects, whereas NO was always performed in the last place, in order to minimize the time required for each experiment.

During each trial, the time needed to complete the three performed tracks was recorded separately. This was done by using a chronometer starting at the time the subject began walking and stopped at the end of each track. Three recordings were thus obtained for each trial and subject.

#### Subjective Analysis

After the completion of the experimental protocol, participants were asked to complete a questionnaire in order to deliver a subjective evaluation of the assistance. The System Usability Scale (SUS) was used consisting of a 10-item questionnaire with 5 options for respondents: from strongly agree to strongly disagree. Subjects were remarked to consider the received assistance itself rather than the load of wearing the device. SUS scores range between 0 and 100 (U.S. Department of Health & Human Services (HHS), 2016). Moreover, previous contributions further provided an adjective scale to better interpret the results from SUS (Bangor et al., 2009). In general, a score in the range [0–25] would correspond to the “Worst imaginable” solution, [25–40] to “poor,” [40–55] to “OK,” [55–75] to “good,” [75–85] to “excellent,” and [85–100] to the “best imaginable.”

#### Data Processing

Group analyses of the kinematic and assistive torque patterns were restricted to steady-state cycles, i.e., when the AO was synchronized to the subject’s movement (phase error at foot strike smaller than 10% of the gait cycle).

First, the performance of the controller was analyzed by assessing the stimulations and assistive torque profiles generated by the controller in the steady-state regime. Second, the influence of the assistive torques on the gait pattern was analyzed. The kinematic profiles were examined together with the joints range of motion (ROM), and the left–right symmetry index. Regarding the symmetry analysis, only the joint angles of both hips were available and were thus compared.

Symmetry of both hip movements was assessed by comparing their respective ROM:

$$SI_{\mathrm{ROM}}=100\left(1-\left|\frac{{\mathrm{ROM}}_{\mathrm{left}}-\mathrm{RO}M_{\mathrm{right}}}{\mathrm{RO}M_{\mathrm{left}}+\mathrm{RO}M_{\mathrm{right}}}\right|\right)$$

With this index, a perfectly symmetrical ROM across both legs would correspond to a symmetry index equal to 100%. By contrast, if one of the legs does not move (i.e., with ROM equal to 0), then **SI**_**ROM**_ would be equal to 0%. Additionally, the gait cadence during each locomotion task—provided as a state variable of the AO—was evaluated.

In order to assess the impact of the assistive trials in the gait behavior and overall performance, statistical analyses were performed on the different trials. Due to the limited size of the population, data could not be proved to be normally distributed. Consequently, Wilcoxon signed-rank test was selected for the analyses. Moreover, a Bonferroni correction was applied to account for multiple comparisons, so that the significance threshold was put to *p* < 0.05/3 = 0.0167 (Holm, 1979; Dagnelie, 2006). This test was performed to check significant changes in ROM, gait symmetry, and gait cadence during the steady-state cycle. A similar analysis was performed on the total time taken to complete the track, averaged over the three track completions. This metric goes beyond steady-state behavior, since it also embraces the cycles during the transition phases.

Finally, the controller behavior during the transitions between the different locomotion tasks was studied. In particular, the number of gait cycles required for the oscillator to resynchronize after a transition was quantified.

## Results

In the following sections, results highlight the performance of the controller and its influence on the gait kinematics and locomotion speed of the participants. A specific section is dedicated to the controller behavior during the task transitions. Finally, the subjective analysis is presented.

The percentage of the selected steady-state cycles taken into account for the pattern analyses with respect to the total amount of cycles is reported in Table 3, for each participant, and for the trials without (TM) and with (AM) assistance.

**Table 3:** **Percentage of steady-state cycles analyzed for each subject and locomotion task during transparent mode (TM) and assistive mode (AM) (%)**.

	Walking		Stair ascending		Stair descending	
	TM	AM	TM	AM	TM	AM
S1	66.3	65.9	71.4	69.1	71.4	50
S2	67.8	73.7	76.2	85.7	76.2	69.1
S3	75.2	74.6	76.2	83.3	76.2	40.5
S4	65.9	73.2	71.4	73.8	71.4	69.1
S5	70.2	66	83.3	85.7	83.3	42.9
S6	66.4	59.4	83.3	78.6	83.3	66.7
S7	65.5	34.8	69.1	64.3	69.1	71.4

### Controller Performance

#### Adaptive Virtual Muscle Stimulations and Torque Profiles

**Figure 4:**
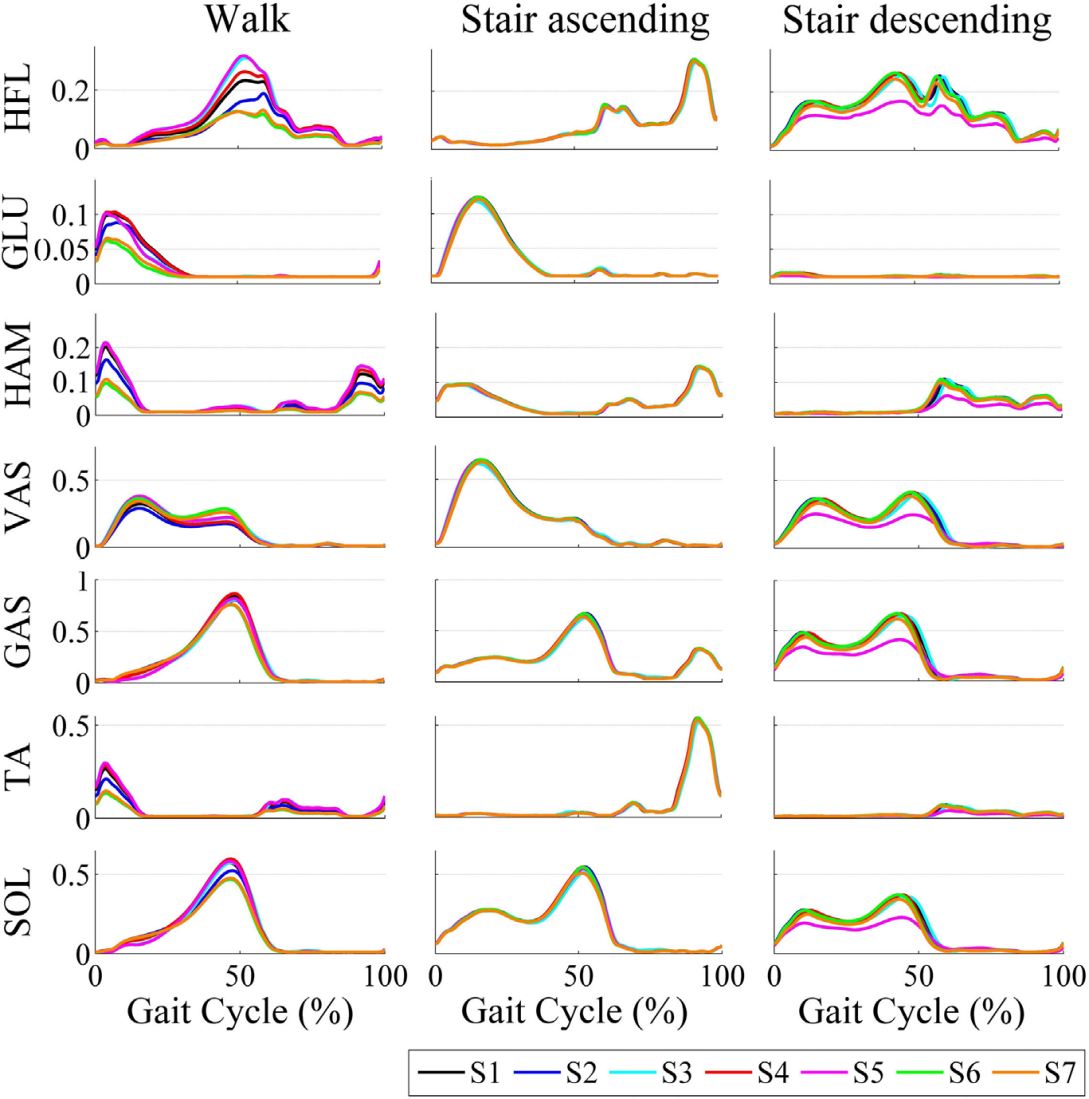
**Averaged stimulations generated by the motor primitives during walking and ascending/descending stairs for all subjects**.

The averaged stimulations generated for the left leg for the different tasks and subjects during the AM trials is depicted in Figure 4. The profiles for ascending and descending stairs display less variability among subjects since they do not adapt to the gait frequency.

**Figure 5:**
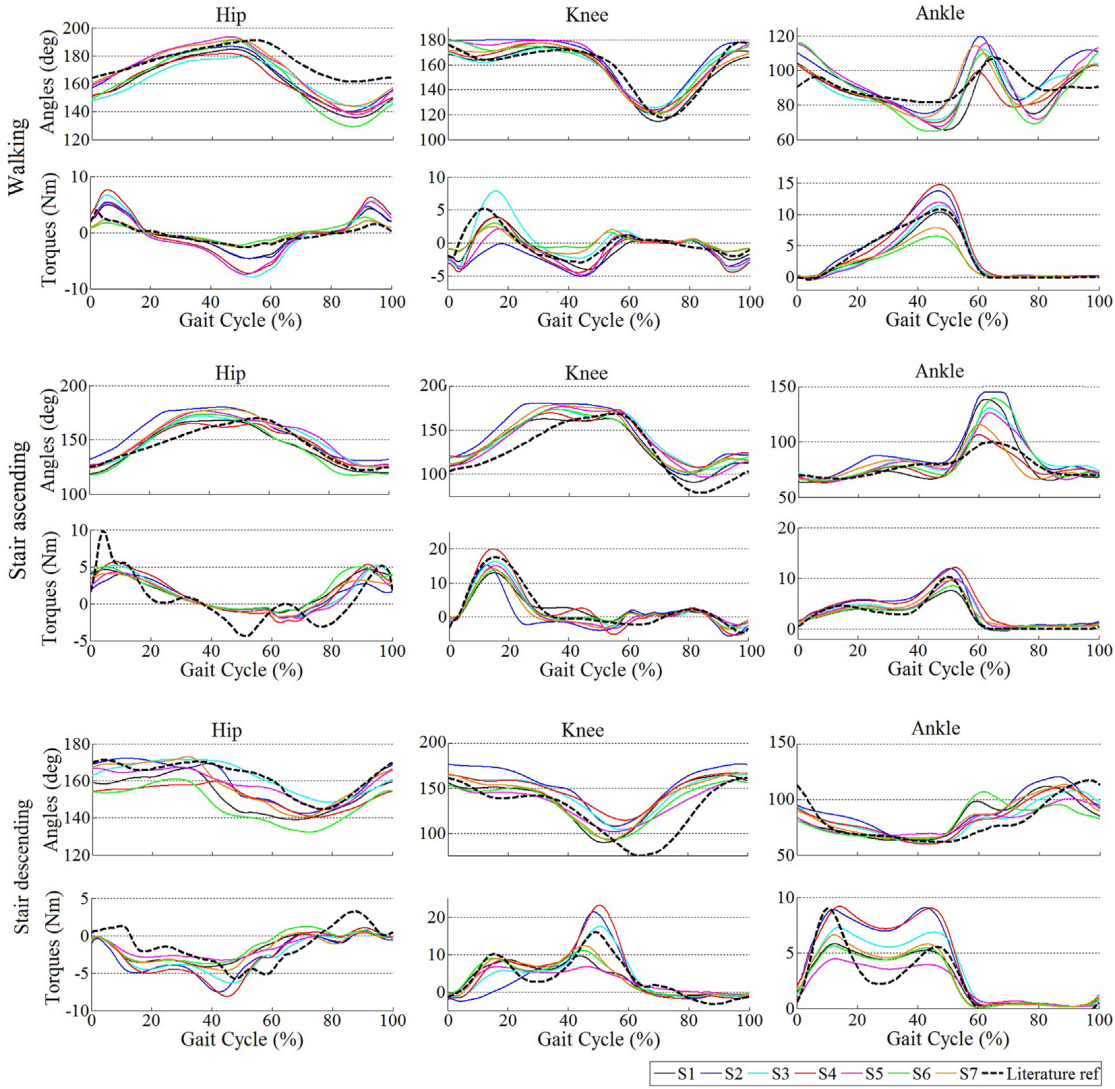
**Averaged kinematics and reference torques for all subjects during the trials with assistance (AM)**. For hip and knee, positive torques correspond to extension and negative ones correspond to flexion. For the ankle, positive torques capture plantar flexion and negative dorsiflexion. In the walking task, the literature reference corresponds to Winter (1991), the torque profiles being scaled by 15, 18, and 10% for the hip, knee, and ankle, respectively. In the stair ascending and descending tasks, the reference corresponds to Bradford and Winter (1988), the torque profiles being similarly scaled down.

Figure 5 shows the mean joint angle patterns and the mean assistive torque patterns during walking, ascending stairs, and descending stairs, again during the AM trials. It also reports two patterns from literature data (taken from those reported in Table 1) as references (Bradford and Winter, 1988; Winter, 1991). An overall consistency among patterns in terms of magnitude and shape can be observed. Moreover, the kinematic profiles are similar to those reported in the literature; and the generated torque profiles follow the same patterns as the torques actually produced by humans during these locomotion tasks (Bradford and Winter, 1988; Winter, 1991). This illustrates that our primitive extraction process did not prevent to generate biologically consistent torque profiles. Moreover, a larger variability in shape and magnitude is visible for the stair descending task than for the walking or stair ascending, both regarding the kinematics and the generated torque profiles.

Additionally, differences in shape and magnitude of the output stimulations and torques for each subject are visible. This shows the adaptability mechanisms of the proposed control to the gait phase, different tasks, and walking cadences, and to the individual locomotion pattern. The particular influence of each of these adaptability mechanisms is further discussed in Section “Discussion.”

#### Adaptation during Transitions between Locomotion Tasks

One of the strong points of the presented control strategy is the use of the same primitives to generate all leg reference torques for several tasks. Modulating these primitives with weights following smooth evolutions from one task to another resulted in smooth transitions of the generated torques.

**Figure 6:**
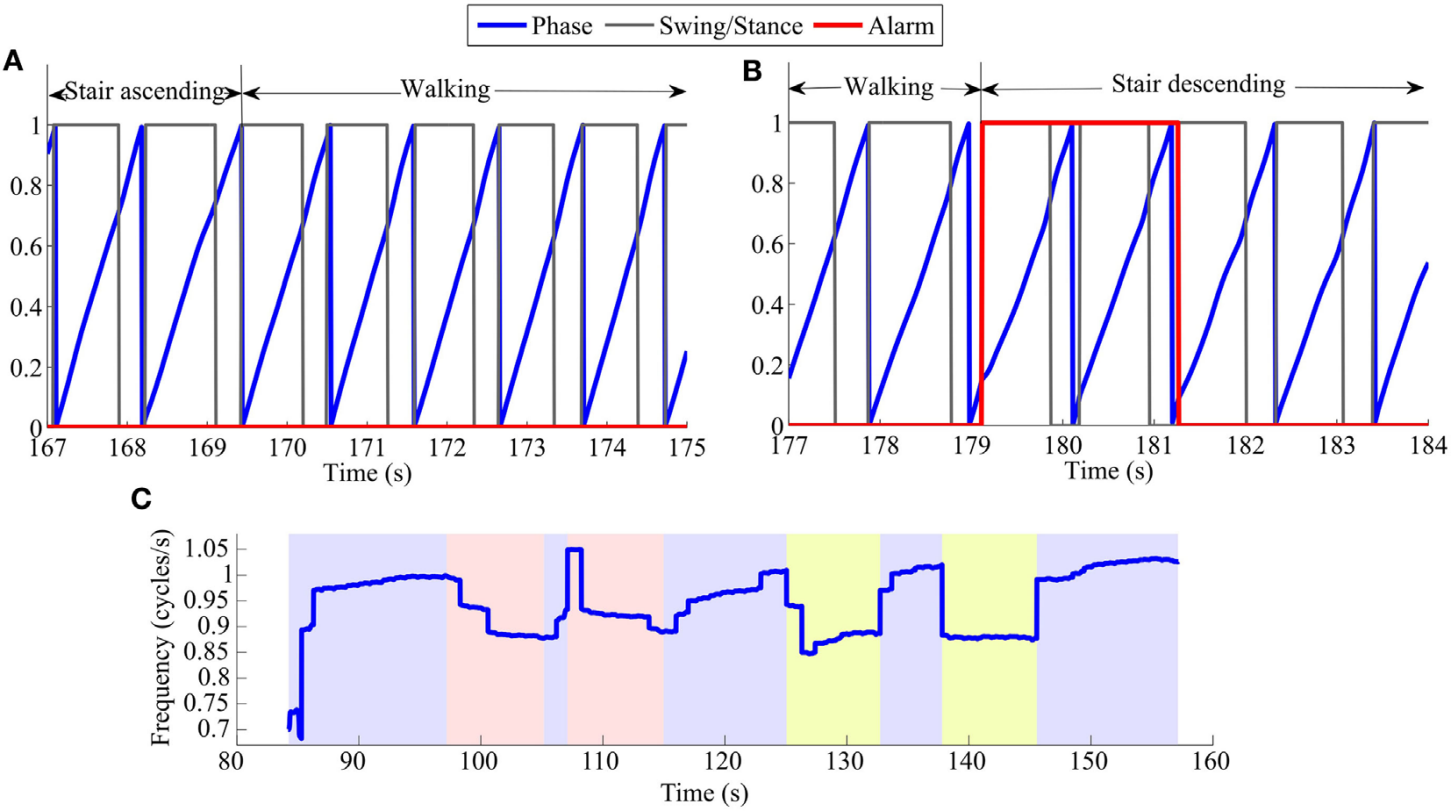
**Examples of phase error detection after task transitions: (A) from stair ascending to walking, (B) from walking to stair descending**. The blue lines capture the actual phase, increasing from 0 to 1 (0–100%). The black lines capture the identification of the swing/stance phases (corresponding to 0/1, respectively). The alarm (red lines) captures the period where synchronization was considered to be lost. It is set to 1 when the phase error at the moment of foot strike is larger than 10% of the detected gait period. **(C)** Frequency in cycles/s for a representative TM trial of S3. The trial is divided in color segments representing periods of walking (blue), ascending stairs (red), and descending stairs (yellow).

However, variations in the hip angle during transitions from one locomotion task to another could cause the oscillator to desynchronize with the gait pattern. Consequently, a few cycles were required to synchronize again, i.e., to re-learn the gait phase and frequency. Table 4 displays the average number of gait cycles taken by the oscillator to drop below 10% of phase error after the four transitions being encountered in our track, i.e., walking to ascending/descending stairs and ascending/descending stairs to walking. This value varied across subjects and trials. In order to guarantee the robustness of this analysis, two different thresholds were used: the first analysis reported the number of cycles before the phase error at foot strike goes below 10% for the first time; and the second analysis considered the oscillator to be synchronized if the error dropped below 10% for more than two consecutive cycles. This second analysis was thus more conservative. Table 4 further shows the group median and the 90% range of data (i.e., between the 5th and 95th percentiles) across subjects. In the best case, synchronization was not lost so that no cycle was necessary to get back to synchronization. In the worst case, the phase error stayed above 10% during the whole new locomotion task. This actually happened only four times across all trials and subjects, always during a transition from walking to stair descending. Figure 6 shows an example of a successful transition (from stair ascending to walking) and another one (from walking to stair descending) in which the oscillator required a few steps to resynchronize. In the latter, the phase detected by the oscillator is larger than 0 (and exceeds 10% of the detected gait period) at the first foot strike after the task transition. Therefore, synchronization was considered to be lost. After two steps, the phase at foot strike drops below 10% and so synchronization is considered to be retrieved.

**Table 4:** **Average number of cycles required to synchronize during transitions**.

	Walk–SA	SA–Walk	Walk–SD	SD–Walk
*S1*	1.67 (1.67)	0 (3.33)	2.67 (2.67)	2.67 (2.67)
*S2*	0 (0)	0 (0)	1.33 (1.33)	1 (1)
*S3*	0 (0)	0 (3.33)	1.67 (3.33)	2 (2)
*S4*	0.33 (2.33)	0 (0)	1.33 (1.33)	0.67 (2)
*S5*	0 (0)	0 (3)	1 (3)	0.3 (0.33)
*S6*	0.33 (0.33)	0 (2.33)	1.67 (1.67)	0.67 (2.67)
*S7*	1 (1)	0 (1.5)	1 (1)	2 (2)
Median	0.33 (0.33)	0 (1.5)	1.33 (1.67)	1 (2)
Rg	[0–1.67] ([0–2.33])	[0–0] ([0–3.33])	[1–2.67] ([1–3.33])	[0.33–2.67] ([0.33–2.67])

The assistive mode trials median and 90% range of data (Rg) are provided at the bottom of the table. Numbers between parentheses capture a more conservative approach (see Section “Data Processing”). SA stands for “stair ascending” and SD for “stair descending.”

**Figure 7:**
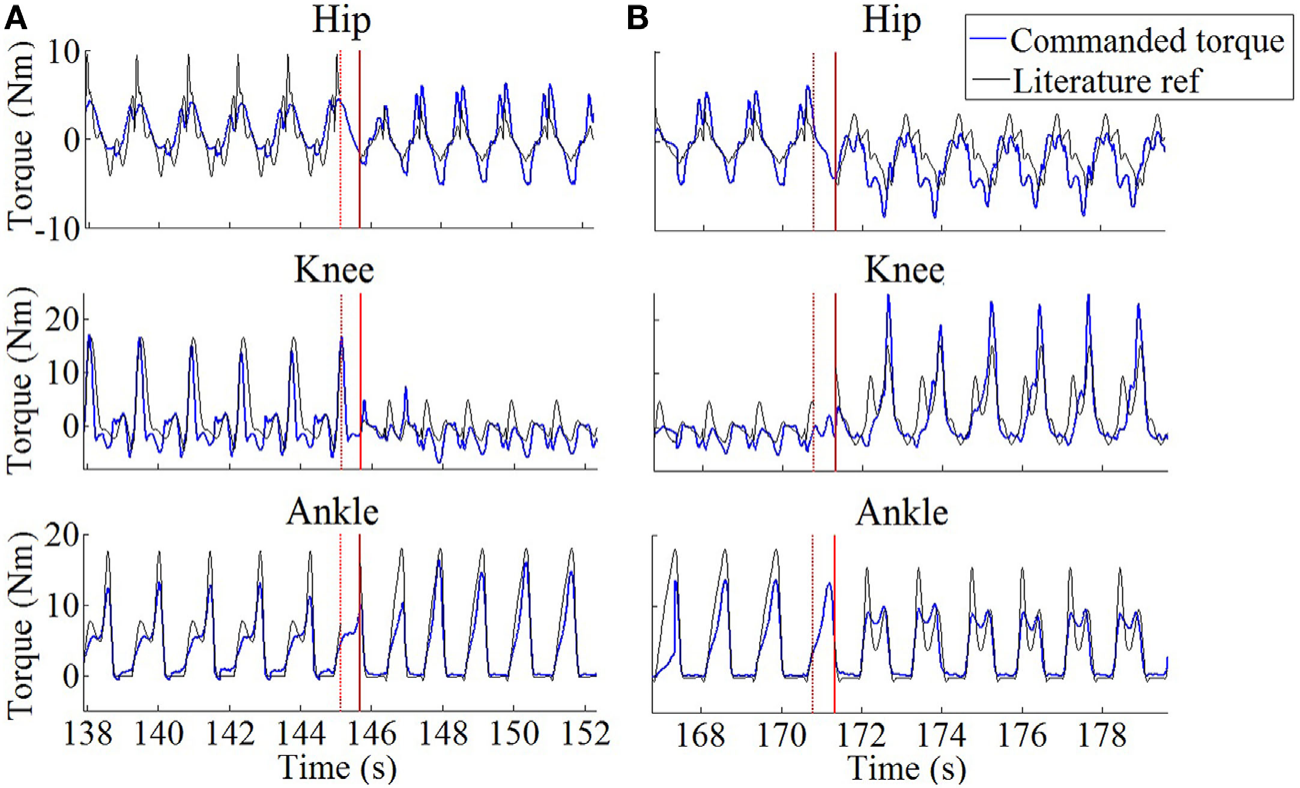
**Generated profiles in the commanded joint torques (blue) during representative task transitions for S2**. **(A)** Stair ascending to walking. **(B)** Walking to stair descending. Black lines are constructed from reference profiles found in the literature [Winter (1991) for walking and Bradford and Winter (1988) for stair maneuvers]. These torques were scaled according to S2’s weight and walking cadence, and to the amount of delivered assistance (see Section “Participants” and “Experimental Protocol”). The red solid vertical lines capture the moment where the locomotion task change was detected. The red dotted lines capture the moment of right toe-off just before this task change was detected.

Figures 7A,B display the generated torques during a representative transition from stair ascending to walking and from walking to stair descending, for *S2*. The figure nicely illustrates the capability of the controller to generate smooth transitions between the different locomotion tasks while reaching the new steady state after a couple of cycles only.

### Gait Changes due to the Assistive Patterns

#### Kinematic Profiles

The following analyses were again restricted to the steady-state cycles. The fraction of these cycles with respect to the total amount is reported in Table 3.

**Figure 8:**
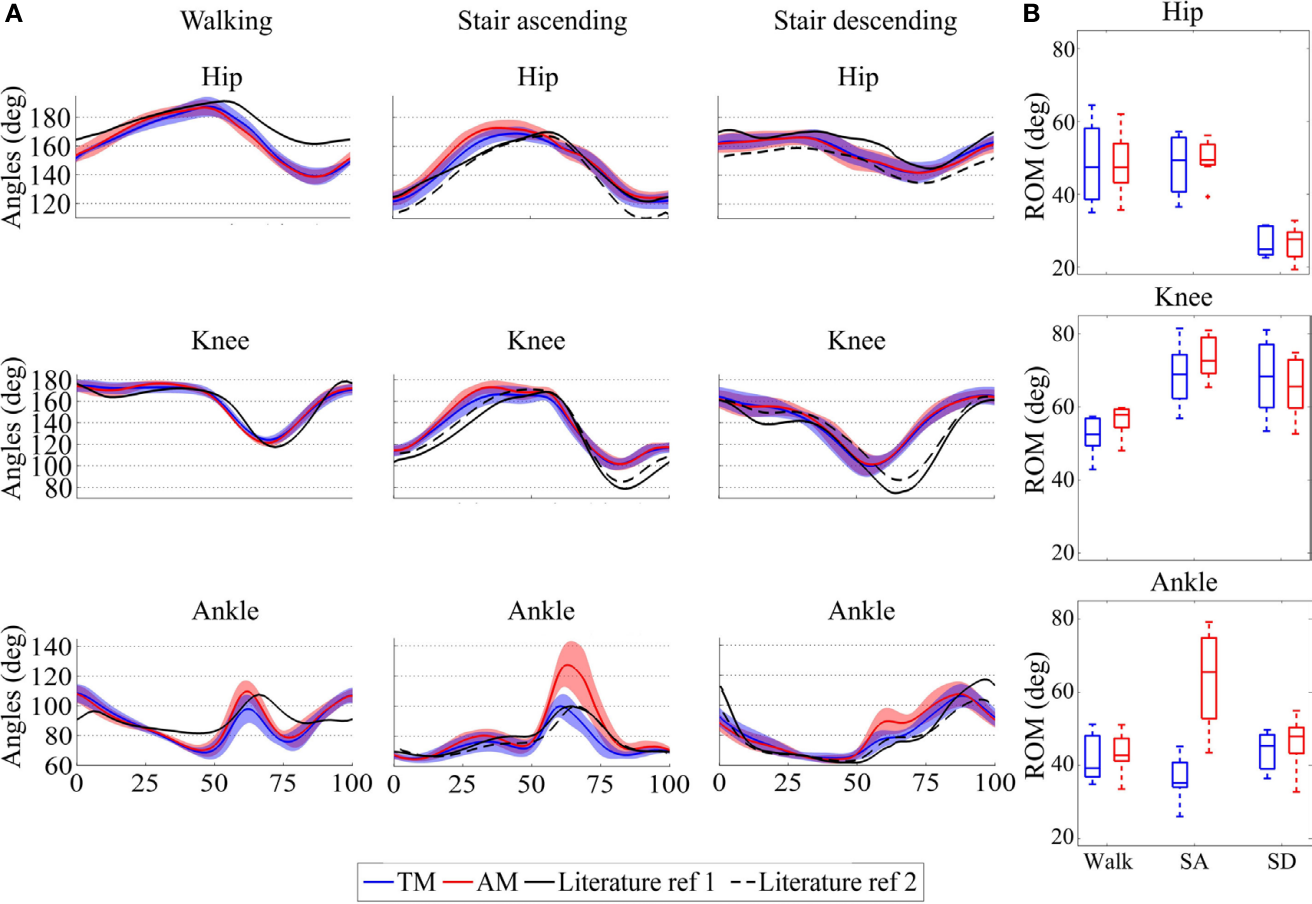
**(A)** Average and standard deviations of joint trajectories across the seven participants for hip, knee, and ankle joints for the three different tasks: walking (Winter, 1991) and stair ascending and descending (Bradford and Winter, 1988; Riener et al., 2002). **(B)** Median, 25th, and 75th percentiles of range of motion (ROM) for walking and ascending/descending (SA and SD) stairs across the seven participants. The different panels report the hip, knee and ankle ROM. The figure reports the patterns in the unassisted (TM, blue) and assisted (AM, red) conditions. For the hip and knee, values above 180° capture extension and below 180° flexion. For the ankle joint, values above 90° represent plantar flexion and below 90° dorsiflexion.

Figure 8A displays the mean joint trajectories of the leg wearing the orthosis compared to references from the literature, in both the TM and AM trials. Again, these trajectories are consistent with those reported in the literature (Bradford and Winter, 1988; Winter, 1991; Riener et al., 2002). More interestingly, the figure reveals that they did not exhibit major changes between the assisted and unassisted trials. One noticeable exception is the increase in ankle plantar flexion angle at push-off, especially for the stair ascending task. In this case, Figure 8 reports both reference patterns taken from literature for stairs (see Table 1; Bradford and Winter, 1988; Riener et al., 2002).

Analyzing the ROM of the three joints without or with assistance provides the averaged group results depicted in Figure 8B, for walking, stair ascending, and stair descending.

At the hip level, the ROM was smaller in the stair descending task than in the other tasks, which is in agreement with reference locomotion patterns (Bradford and Winter, 1988; Winter, 1991; Riener et al., 2002). However, there was no significant difference in the hip ROM between the TM and AM trials (*p* = 0.94 for walking and *p* = 0.58 for stair ascending/descending), variations in ROM being smaller than 3°.

The knee ROM increased by 5.42° and 3.7° in the walking and stair ascending tasks, respectively, when assistance was delivered. It decreased by 2.77° in the stair descending task. Again, no statistical significant difference was found between TM and AM (*p* = 0.078 for walking, *p* = 0.031 for stair ascending, and *p* = 0.30 for stair descending). Finally, statistics confirmed the observed increase by 30.35° in the ankle ROM for the stair ascending task (*p* = 0.016), whereas no significant difference was found for walking or stair descending (*p* = 0.22 and *p* = 0.16, respectively) which changed by less than 4°.

A further point of analysis was the impact of the assistance on the gait symmetry. Table 5 shows the median of **SI**_**ROM**_ and the 90% range of data across subjects among the different locomotion tasks. Values in both trials were of similar order and stayed above 90%, showing a good symmetry between the ROM of both hips. Consistently, no statistical difference was found between TM and AM (*p* = 1, *p* = 0.69, and *p* = 0.81 for walking, ascending stairs, and descending stairs, respectively), revealing that the assistance did not introduce any significant change regarding gait symmetry, at least at the hip level.

**Table 5:** **Median and 90% range of data (Rg) of Symmetry index of the range of motion**.

	Walking		Stair ascending		Stair descending	
	Median	90% Rg	Median	90% Rg	Median	90% Rg
Transparent mode (TM)	94.1	[80.1–98.3]	95.4	[84.8–99.5]	91.3	[82.6–99.1]
Assistive mode (AM)	92.9	[75.8–99.2]	93.8	[84.7–98.1]	93.4	[83.3–98.1]

#### Cadence and Speed Adaptation

Complementary analyses were performed to evaluate the influence of the assistance on the gait cadence (frequency), which is provided as an output of the AO. An example of the oscillator estimation of the gait frequency over one full track under the TM condition is displayed in Figure 6C.

Table 6 displays the individual and group median gait cadence (with 90% range of data) during both TM and AM trials and for the different locomotion tasks, restricted to the steady-state cycles. A global trend suggests an increase of the gait cadence when assistance was delivered, ranging between 4 and 10% depending on the locomotion task. However, statistics reached significance only for the walking task (*p* = 0.016), and neither for the stair ascending nor for the descending ones (*p* = 0.031 and *p* = 0.16, respectively).

**Table 6:** **Cadence (cycles/s)**.

	Walking		Stair ascending		Stair descending	
	TM	AM	TM	AM	TM	AM
S1	0.83	0.90	0.67	0.70	0.69	0.67
S2	0.79	0.81	0.70	0.73	0.68	0.76
S3	0.99	1.02	0.93	0.95	0.88	0.99
S4	0.89	0.94	0.77	0.88	0.76	0.93
S5	0.99	1.02	0.90	0.95	1.03	1.08
S6	0.66	0.66	0.61	0.62	0.65	0.63
S7	0.68	0.69	0.61	0.60	0.57	0.58
Median	0.83	0.90	0.70	0.73	0.69	0.76
90% Rg	[0.66–0.99]	[0.66–1.02]	[0.61–0.93]	[0.60–0.95]	[0.57–1.03]	[0.58–1.08]

*Median across subjects and 90% range of data (Rg) are provided at the bottom of the table*.

Finally, we also measured the time needed to perform the track (Table 7). This measurement was the only one being also accessible in the NO trials. In general, wearing the device induced a strong penalty in the track completion time, with an average increase of 30% between TM and NO. The track was however completed faster when assistance was delivered than in the TM condition. This decrease corresponded to about one third of the penalty caused by wearing the device.

**Table 7:** **Total time needed to complete the track(s) for every subject**.

	NO	Transparent mode	Assistive mode
S1	89.1	141.4	134.7
S2	76.3	134.4	125.3
S3	89.8	111.6	106.7
S4	88.5	123.6	104.2
S5	85.9	100.4	93.2
S6	77.7	100.3	98.4
S7	76.7	103.3	101.6
Median	85.9	111.6	104.2
90% Rg	[76.3–89.8]	[100.3–141.4]	[93.2–134.7]

*Median across subjects and 90% range of data (Rg) are provided at the bottom of the table*.

Statistical analysis was again performed by individually comparing trials. Significant difference was obtained between each pair of trials: significant increase from NO to both other conditions (*p* = 0.016 in both cases) and a significant decrease from TM to AM (*p* = 0.016). Therefore, the assistance did contribute to decrease the burden of wearing the device, yet not enough to overcome the whole penalty caused by the device itself.

#### Subjective Analysis

After completing the SUS questionnaire, scores were 70, 80, 75, 75, 65, 65, and 57 for each subject, respectively. According to the adjective scale, this means that four subjects considered the assistance as “good,” while three regarded it as “excellent.”

Interestingly, smaller subjects were the ones giving the less favorable evaluation. In particular, *S5* and *S7* did not fit very well in the APO, which was mainly designed for bigger users.

## Discussion

In this contribution, we presented an experimental validation of a biologically inspired controller based on motor primitives. This controller was derived in order to provide assistance through a leg exoskeleton for the execution of different locomotion tasks.

The paper reported an experiment where participants walked at their preferred speed and with their preferred pattern along a track comprising different locomotion tasks (walking and stair climbing/descending). During the track completion, motor primitives generated virtual muscle stimulations that were modulated as a function of the detected locomotion task, gait phase, and gait frequency (this latter only in the case of walking). Stimulations were then injected into a virtual musculoskeletal model in order to generate muscle forces that were further translated into joint torques. A fraction of these torques was finally delivered at the level of the active joints of the left leg, through a wearable exoskeleton.

### Controller Performance

Motor primitives generated virtual muscle stimulations that entered a musculoskeletal model computing desired assistive joint torques. This motor primitive-based controller featured several advantageous levels of adaptability.

#### Adaptation to the Gait Phase, Cadence, and Locomotion Task

First, an AO was used to synchronize to the user gait pacing, similarly to what has been done in previous research (Ronsse et al., 2011). Consequently, the “phase zero” of the primitives was synchronized to the foot strike, and the primitives duration was scaled on the one of the gait cycle. This was a desirable feature for the subjects, who were not forced to a fixed pace, but could adopt their preferred one and continuously change it during the task completion.

Second, depending on the locomotion task (walking, ascending/descending stairs) and on the walking cadence, the controller modulated the weights combining the set of primitives, in order to provide a torque pattern being relevant for the task and cadence. These modulations gave rise to stimulations which changed in a smooth but agile way between conditions. Interestingly, the intention detection method used in this experiment in order to determine the performed locomotion task was different from the one used in our former publication (Ruiz Garate et al., 2016a). This illustrates the flexibility of our primitive-based approach to be used with different intention detection modules.

Within the same task, Figure 4 illustrated the between-subjects variability. As an example, during walking, *S6* and *S7* generated lower stimulations in magnitude than the other participants. This is consistent with the fact that they displayed lower walking cadences (see Table 6), so that smaller weights were generally used for the recombination of the primitives (see Figure 2). Consequently, all subjects did not receive the same assistive torques, both in magnitude and pattern (see Figure 5). In particular, *S6* and *S7* again received lower torques than the other participants, as a direct consequence of their lower walking cadence (see Figure 5).

Although several cadences were used for building up the primitive weights corresponding to the walking task, a single cadence was used to compute the primitive weights in the stair ascending and descending tasks. Consequently, the current level of development of our controller does not provide pattern adaptations as a function of the gait cadence when taking stairs, both up and down. Compiling a larger amount of literature data reporting different cadences would be necessary to offer this adaptation to different cadences.

As explained in Section “Experimental Setup,” we used one oscillator for tracking the hip angle of one leg to retrieve the gait phase and frequency. The parameters for the other leg where considered to be symmetrical. Using our controller with patients displaying asymmetrical gaits would require to implement one oscillator tracking the movement of each different leg, thus giving the possibility to provide non-symmetrical torque assistance. If the controller is envisaged to be used with patients having no remaining leg movement capabilities, the oscillators could be pre-set to a desired cadence and could be augmented with coupling mechanisms to generate the appropriate left/right anti-phase pattern. Another option would be to obtain the gait phase and frequency parameters from another cyclical movement, like, for example, the swinging of an arm. Note, moreover, that using our controller with patients having no remaining leg movement capabilities would induce extra challenges being out of the scope of the present paper, e.g., the design of a proper leg impedance model or the external management of the task transitions.

#### Handling Transitions

The possibility to execute smooth transitions outperforms existing exoskeleton controllers, as no intermediate stop state is required (Esquenazi et al., 2012). Indeed, subjects can fluently move from one locomotion task to another. However, the synchronization of the AO from one task to the next eventually took several cycles during which subjects received a reduced assistance. This was particularly true when switching to the stair descending task (see Table 4). This observation was likely due to a significant decrease in speed (and gait cadence) during the few walking cycles preceding the stairs, while speed and cadence increased back during the first descending steps. This oscillation in gait parameters negatively impacted the settling time of the oscillator. It is forecasted that a longer familiarization with the device and tasks would allow subjects to perform transitions more fluently, thus reducing the synchronization time. Again, countermeasures should be taken to tackle this problem if the controller is being used with patients. A possibility would be to force the oscillator to follow the phase and frequency being interpolated from the period between the two preceding foot strikes.

Moreover, in this experiment, a single sensorized insole (placed under the right foot) was used to segment swing/stance phases and measure the gait cycle duration. Consequently, when the task transition was initiated with the left leg, there was a half-cycle delay until the task change could be detected. This delay caused no significant perturbation, since our healthy participants were capable of continuing the task despite receiving inappropriate torque profiles during this short amount of time. Figure 7 confirms the matching between the provided torques and the profiles taken from the literature and used to construct our primitives (see also Figure 5). Moreover, it shows that the torque profiles followed a smooth blending in the period between both tasks, i.e., the period between the last toe-off of the right foot in the task before the transition, to the right foot strike in which the task change was detected (about 0.55 s of time difference in both cases). It is worth to note that torque profiles were smoothly delivered as a sequence of flexion/extension peaks (for hip and knee joints) and plantar/dorsiflexion peaks (for the ankle joint), even if some delay was faced in the task detection. We expect thus that such a smooth behavior would induce a minimal interference with the natural human biomechanics. Future developments require placing an insole below each of the feet, in order to deliver bilateral assistance to potentially disabled subjects. This bilateral detection would increase the dependability of the system being the task recognition updated at each step and not at each stride.

Additionally, the intention detection method (see Section “Intention Detection”) would require adaptations for subjects affected by mild lower limb impairments and who are not able to benefit from the provided torque. One possibility would be to trigger a change of locomotion intention with some dynamic movement from the torso or head, or with a similar system as the wrist-pad used in ReWalk (Esquenazi et al., 2012; Murtagh, 2015), or to augment the intention detection algorithm with inputs provided by kinetic signals.

#### Adaptation to Subject Anthropometry and Individual Gait Pattern

Finally, the musculoskeletal model embedded a third level of adaptation of the torque pattern, this time as a function of the particular subject anthropometry and adopted locomotion pattern. For example, *S5* had a slightly higher walking cadence than *S4*, generating higher stimulation magnitudes (Figure 4). However, *S4* was considerably taller (see Table 2), so that the muscle force for the same input stimulation and kinematics was larger, therefore generating larger joint torques (Figure 5). This illustrates how, even if the same level of assistance is selected, different torque magnitudes are produced, as a consequence of the musculoskeletal model adaptation to each individual anthropometry. Simple adjustments to individual anthropometry were performed, as reported in Section “Motor Primitives.” These simple rules would likely have to be revised with elderly subjects and patients for whom the correlation between height and the muscle strength is less evident.

Dependence between the reference torque and the adopted kinematics pattern can also be noticed in Figure 5. For example, during walking, *S2* received the lowest knee extension moment at the beginning of the gait cycle, despite being the second tallest subject and adopting a faster walking frequency than *S6* and *S7* (see Table 6). However, this subject exhibited no knee flexion after foot strike. Flexing the knee caused the virtual VAS muscle to increase in length, while the antagonist HAM and GAS decreased. Consequently, the musculoskeletal model caused them to increase/decrease their respective forces. Moreover, flexing the knee caused the VAS projected force to increase the applied knee extension torque, due to the adaptation of its lever arm (Geyer et al., 2003). In sum, *S2* received no extension torque, since he kept his knee straight after foot strike. In the stair ascending task, a similar observation can be reported for the same subject, between 25 and 50% of the gait cycle (see Figure 5). This illustrates a desirable adaptation due to our musculoskeletal model, i.e., that assistive knee extension torque was only provided if the knee flexed after foot strike.

In sum, larger muscle forces were generated for taller subjects, and these forces were differently mapped to joint torques as a function of the actual kinematic pattern of the subject. This adaptation is particularly interesting with respect to strategies imposing a particular pre-recorded gait trajectory (Suzuki et al., 2007; Lenzi et al., 2013), mainly for two population groups: elderly people, who exhibit a decreased ability to adapt to imposed patterns and devices; and patients with gait deficits, who will not be abruptly forced to follow an “ideal” gait pattern.

### Impact of the Assistance on the Locomotion Behavior

The generated reference torques were scaled as a function of the desired level of assistance being desired. In this experiment, we selected 15, 18, and 10% for the hip, knee, and ankle joints, respectively, as the outcome of pilot tests. This assistance had no significant impact on the gait kinematics, which remained within physiological normal ranges, and were consistent with those being reported in the literature (Bradford and Winter, 1988; Winter, 1991; Riener et al., 2002). A noticeable exception to this statement was the increment in plantar flexion around toe-off during the assisted stair ascending task (Figure 8B). This likely reveals that subjects benefited from an increased push-off moment to propel themselves upstairs. Further tests are necessary in order to evaluate if larger assistive gains could increase the benefit for the users and cause larger changes in the kinematic patterns.

By contrast, providing assistance had a significant impact in the gait cadence, at least for the walking task. Moreover, a significant difference was found when measuring the global time needed to complete the track between the conditions with and without assistance. Actually, all subjects performed the track in a faster way when being assisted (see Table 7). Therefore, the general increment in cadence correlated with the increment of speed, revealing that assistance encouraged subjects to walk at a faster pace. However, assistance did not fully compensate the payload of wearing the device itself. This result is similar to other contributions showing a metabolic increase while wearing an exoskeleton device, which can only be partly compensated with active assistance (Valiente, 2005; Ruiz Garate et al., 2016a). These results are nevertheless encouraging, since better performance can be forecasted with a reduction of the exoskeleton weight. Also, incrementing the assistance level for some tasks can potentially enhance the performance.

Interestingly, all subjects considered the assistance effectively helping them to perform the track (from “good” to “excellent”). This was evaluated by a subjective analysis relying on a SUS questionnaire. Importantly, this reveals that participants felt assisted in their locomotion tasks. Moreover, most of them indicated feeling the difference in the assistive pattern being received when performing the different tasks. This tends to illustrate that the primitive-based assistance being elaborated in this paper can also be perceived at a subjective level by the person wearing the device.

### Future Work

Future experiments should test the possibility of providing different levels of assistance in a task-dependent way, so that the level of assistance of the tasks providing a smaller total reference torque might be increased. Moreover, the described approach should be transferred and tested with a bilateral leg exoskeleton, in order to deliver a similar assistance to both legs. This would require processing the signals from the same leg as the one being assisted for achieving the synchronization process (see Section “Experimental Setup”).

Last but not least, future perspectives will focus on testing the proposed approach with gait impaired subjects to further assess the assistive potential of the controller and better exploit its adaptation capabilities.

## Conclusion

In this paper, a bio-inspired controller based on motor primitives was developed for human locomotion assistance and validated through a series of experiments. The controller modulated the same set of primitives to generate torque references during ground-level walking and stair ascending/descending for the hip, knee, and ankle joints.

The controller proved to adapt to these intrinsically different locomotion tasks and generated smooth transitions between them without significantly perturbing the locomotion pattern of healthy subjects. Moreover, delivering the assistance helped the participants to significantly reduce the time needed to perform the track, though not to the point of compensating the burden due to the weight of the device itself.

## Author Contributions

VRG, AP, TY, NV, and RR significantly contributed to the development of the experimental methods, the execution of experiments, the analysis of results, and the edition of the manuscript. MM and RML significantly contributed to the development of the experimental methods and the edition of the manuscript. All the authors approved the submitted version of the manuscript.

## Conflict of Interest Statement

The authors declare that the research was conducted in the absence of any commercial or financial relationships that could be construed as a potential conflict of interest.

## Footnotes

^1^“Foot strike” is used instead of “heel strike” because locomotion tasks such as stair descending involve toe contact before heel contact.
